# Where Do We Fit? Reflections on Research Interview Practice, Project Design, and Interpretation**

**DOI:** 10.1002/bewi.70010

**Published:** 2026-02-24

**Authors:** Dmitriy Myelnikov

**Affiliations:** ^1^ Department of History and Philosophy of Science University of Cambridge Free School Lane Cambridge CB2 3RH UK

**Keywords:** oral history, research interviews, triangulation, historiography, historical fieldwork

## Abstract

What is special about historical research interviews in the history of science, technology, and medicine, and how do they compare to the tools of oral historians and social scientists? This essay reflects on three interview projects I have undertaken, each taking a distinct shape. I address methodological and pragmatic aspects of these projects, especially concerns related to anonymity and recovering memories of practice, as well as institutional challenges for placing historical research interviews among the more familiar and structured projects in the social sciences, notably around questions of anonymity. I suggest that we have most to learn from oral historians in thinking about research ethics and empowering the interviewee to decide what they want to happen to the recording and transcript, and whether and how they want to claim credit. At the same time, we can make different choices when it comes to designing, interpreting, and publishing our interviews. I suggest it may be beneficial to think of our work as triangulation across sources, within broader practices of what we might call “historical fieldwork”.

## 1 Introduction

For a historian of science, technology, and medicine, methodology can be an uncomfortable word. It is something we normally investigate in the natural sciences and beyond, something constructed, adopted by others, and sometimes contested. In our own work, I prefer to think of approaches and themes, and on a more mundane level, of ways of interpreting individual sources—often very specific and idiosyncratic, sometimes rather generic. The use of interviews is perhaps the most significant case where bigger‐picture questions of methods arise, both in planning and conducting the work, but also in justifying its design to colleagues, funding bodies, and ethics review committees. In this essay, I draw on my experience conducting interviews in the UK and the United States, while based at British universities, for three projects with different design problems: interviewing scientists and biotech entrepreneurs for the history of genetic engineering in mice; a collective interview following the witness seminar format among many of the key players in the cloning of Dolly the Sheep; and a project on the history of animal research in the UK that involved speaking to a wide range of professionals, including veterinarians, activists, and civil servants, but primarily focused on animal technologists who care for lab animals in dedicated facilities.^1^

My training has come from oral historians (the Oral History Society in the UK, including the Wellcome Trust–funded five‐day course they used to run),^2^ but I have also interacted closely with social scientists working in science and technology studies who rely on a variety of methods: most relevant here, qualitative interviews and ethnography, but also surveys, including via the Mass Observation Archive (also familiar to some British historians),^3^ artistic practice, and creative public engagement. While I haven't personally conducted any of this work (except a hybrid interview covering both historical and contemporary practice, with a sociologist colleague), it has been very helpful in reflecting on my methods and differences in approach.

The boundaries of the term “oral history” have been somewhat blurry. In broadest terms, as a discipline, it concerns itself with the compilation and study of recollections, usually using recorded interviews with people having personal knowledge of the past. As a discipline and methodology, oral history has roots in the impetus to write history from below, with social historians’ interests in local communities, their domestic and working lives, which had been ignored by mainstream historians. It has since had many uses, in other areas of social history focusing on gender, race, and sexuality, in museums and heritage, and in broadcasting. More recently, oral history has been adopted in popular culture journalism, both written and in podcast form.^4^ With an increased focus on indigenous histories, decolonizing knowledge practices, and thinking about ways of knowing beyond Western science, the older, overlapping meaning of oral history—collective unwritten knowledge of past events passed through speech—has received new salience, while building on long traditions of research and activism.^5^

Soraya de Chadarevian has argued that, in the context of the history of science, research interviews are distinct in their aims and approach from oral history and are best thought of as a collaboration between the interviewer and interviewee. The outcomes will vary depending on interview conditions and timing, respective levels of knowledge about the subject matter, rapport, and contingencies of conversation.^6^ Even in the 1990s, however, some oral historians recognised the collaborative process, and much more so since then, with emphasis on inter‐disciplinarity—oral history is now a broader church.^7^ Still, the approach many historians of science, technology, and medicine embrace is distinct from the established tradition of open‐ended and extensive life story interviews, covering several sessions of several hours. The kinds of methods I used relied on short interviews of about one or two hours, semistructured or unstructured, in that I had a series of questions and topics I wanted to cover, but was largely happy to let the interviewee speak and take things in a direction they wished. It was, for me, one tool and set of sources among many, and oral testimony was not the central aspect of these projects.

Paul Merchant has called on historians of science to take on board the oral historian's sensitivity to interpreting interview narratives—something historians of science do routinely apply to textual sources. He also suggested that oral historians, much of whose mainstream work has recently focused on processes of personal and narrative composure in life stories, might learn from historians of science using interviews as a source to primarily illuminate the past and cast new light on events, closer to the initial social‐history ambitions of oral history itself.^8^ My intervention, written with historians of science and medicine, seeks to make both more practical and more general points. Building on Merchant's call, I suggest that when conducting research interviews, we have the most to learn from oral historians, both by taking inspiration from the kind of insights extensive engagement with long‐form interviews can generate,and when it comes to the craft of interviewing, interpreting the recordings, and the ethical choices one faces throughout the process. In most cases, however, the work we do will involve other sources, both traditional and novel, and constructing historical narratives across them, from a selective and often contingent patchwork we have available. To suggest a way of navigating through these sources, I draw on the metaphor of triangulation used by social scientists (and increasingly, historians and philosophers of science). More generally, this essay seeks to celebrate and encourage historians’ promiscuous approach to methodology—borrowing methods, techniques, and ways of thinking from other fields, and adjusting them to our specific interests, bodies of work, and traditions.

## 2 Project Design: The Ethics of Anonymity

A major distinction between (most) social science and historical qualitative research is the question of anonymity. Ethnographic and social research projects often default to blanket anonymization, where all identifying information is stripped from notes and transcripts, and much of the hands‐on methodological discussion revolves around robust but realistic strategies, especially in the digital world.^9^ Since research usually involves contemporary practice and groups, often potentially vulnerable, confidentiality becomes a key ethical responsibility. Oral historians, however, do not default to anonymising. In interviewing scientists or other elite groups, it often does not make sense to anonymise—in most cases, doing so would eliminate context relevant for historical interpretation. It can also be very challenging to achieve reliable anonymity, especially in small research fields. But more fundamentally, in part because of its history‐from‐below origins and emphasis on giving agency to people whose lives may not have generated extensive records, oral history methods strongly recommend giving names to narratives unless there are compelling reasons not to. The choice is best left to the interviewee as part of the conversation around informed consent and copyright.^10^

In my experience of ethical review in the UK university system, the question of anonymity is what caused tensions when it came to project design, and challenge from social scientists, and (rightly) increased levels of scrutiny. Having animal technologists go on record raised the greatest concern. Since the intensification of animal rights activism in the UK and elsewhere in the 1970s and 80s, including direct action such as laboratory break‐ins, working in animal research raised the levels of safety concerns. Several of my interviewees had received hate mail and threats, which included publicising their work among their neighbours and communities. In other extreme cases in the UK, there have been bomb attacks and targeted criminal harassment.^11^ Many noted that while they had been able to talk about their work freely in their communities before the 1970s, secrecy and euphemisms became the default in later years—patterns also observed in other countries, notably the United States and Germany.^12^ Universities and research institutes, as well as industrial laboratories, upped security, and animal facilities adopted ambiguous names such as “biological services.” Professional meetings were being conducted in secrecy, with last‐minute reveals of location. The situation is less intense than it had been in the 1990s, with a crackdown on extreme animal activism by the Blair government in the early 2000s and an increasing culture of openness relieving some tension, but the concern was real.^13^

With all of that in mind, anonymising by default would seem the responsible thing to do. Yet my colleagues and I felt forced anonymisation, even in a case such as this, had its own ethical dangers, not least scholarly paternalism in assuming lack of credit was justified by our concerns.^14^ In the end, in line with best practice advice from the Oral History Society,^15^ we decided to extend the decision to our interviewees, giving them the option to change their minds up to the point of publication or deposit, with extra checks when using quotations. In this case, as far more experienced in dealing with potential risks involved in their work, the interviewees were best placed to make this decision. A few chose to remain anonymous, but many did not.

Anonymity can be an ethically charged issue, and at times its provision can be essential for interviewees to trust the project and the process. Power relationships—across class, gender, race, and education—can become a major concern, and these are often messy, especially at an intersection of social science research, participatory research, and oral history. As Lisa Bourke's analysis of several participatory research projects with Australian Aboriginal communities shows, confidentiality was a central concern for most interviewees when it came to health issues, such as breast cancer.^16^ More recently, Calabria and Bailei have stressed leaving anonymity up to participants even in cases where sensitive information—in their case, mental health diagnoses—is being discussed.^17^ Ethical and legal obligations must guide decisions; in this instance, EU GDPR legislation forbade publication of materials where third parties' mental health diagnoses were revealed, and those interviews were embargoed. This case, however, is a reminder that full anonymisation is not the only way to safeguard individuals; embargoes and limited access are other options, with future historians potentially able to access the information that has been preserved with its full richness.

## 3 The Encounter

What the individual interviewer brings to the conversation shapes the interview, and indeed whether it happens in the first place, and on what terms. My undergraduate training in biology has influenced many conversations, especially with life scientists and medical researchers. While their expertise and knowledge were of course much greater than mine, this background influenced the questions that I asked and the level of response, resulting in occasional esoteric passages relevant to my interest in laboratory practice and technique. In these exchanges, something must have inevitably been lost, too—not least a deeper level of reflection when one is invited to approach one's practice from a less familiar perspective.

One's commitment, background and institutional position can also determine who chooses to participate. In the charged area of animal research, the team's connections and my scientific training helped us build rapport with senior animal technologists and laboratory veterinary surgeons, especially those actively involved in professional networks and feeding into the policy process. At the same time, however, some (but not all) activists and members of antivivisection groups have treated my approach with suspicion, and we managed to conduct very few interviews among them. While there was a diversity of opinions on animal research within the project, the ambition to integrate humanities and social sciences into open conversations about the issues of experimentation and welfare meant engagement with its practitioners, which in turn raised suspicion among people with stronger abolitionist commitments. This of course shaped the research questions and sources, and I relied instead on government documents and correspondence, media reports, ephemera, and secondary sources, but it left my understanding of activism against 1980s reform of UK animal experimentation more opaque than it might otherwise have been.

Oral historians have moved on from the notion of a professional, remote, nearly invisible interlocutor to recognise the interview as a co‐construction, perhaps collaboration, between the oral historian and the interviewee.^18^ Recognising the contingency of an interview does not, however, eliminate the practical questions of technique. The multitude of methods honed and elaborated by oral historians, as well as social scientists, ought to be taken seriously.^19^ Leading questions give predictable and potentially misleading answers. Active listening is important, and it can become tiring. Following up on what the interviewee wants to talk about or brings up unexpectedly can be more valuable than following a rigid questionnaire—there is usually time to cover the key questions. It is worth leaving the questions open; the interviewer should not rush to fill the silence but rather ignore the discomfort and leave space for the narrator to say more if they wish. These techniques do not have to be about removing the interviewer from the narrative; rather, they help produce a better conversation and a richer account.

What is covered and how things get framed depend on so many factors: time, location, rapport, and how quickly and well it is established. Some research subjects who have been interviewed many times, by historians or journalists, may have a stable, almost rehearsed narrative of their life and career, but that can present problems in moving beyond those fixed accounts, especially if time is limited.^20^ In another case, a prominent scientist who had already been interviewed by another scholar was suspicious about having another interview once the narrative of their career was already established, and weary of introducing contradictions. In all these conversations, subtle and intersecting dynamics of race, ethnicity, gender, age, dis/ability, and class may shape and channel what is and is not said. Thinking through these problems highlights the implausibility of removing the interviewer from the product, which is, after all, a record of a conversation.

## 4 Interpreting the Interview

Interviews stand out in reconstructing collective memory of events, disciplines, or research agendas, often showing tensions between contemporaneous records and practices of memorialisation. The distinction some historiographers have drawn between the historical and practical past—the former more academic and concerned with past's own terms, the latter perhaps less consistent with archival sources but important and poignant to communities in the present—is relevant here.^21^ Historians of science have certainly been interested both in how scientists present their work and its history, and have sought to contribute to current debates on the past.^22^ Except for some key witness seminar projects, such as the Wellcome Trust series, it is curious that some of the key scholarly works on scientific memorialisation—the scientists’ practical past—have not relied on oral history, even where practical, but rather published and archival sources such as *Festschriften*, journal reporting, and records of commemorative events.^23^

The witness seminar is, moreover, a reminder of the long‐established diversity of approaches within oral history. The format brings its own limitations and choices that shape the discussion greatly. Transactive memory, or the communicative act of remembering in groups, can be valuable but also hinder individual memories, or colour accounts in ways that might be quite distinct from recollections in a one‐on‐one interview.^24^ Selecting participants, the contingencies of who is available and willing to participate, decisions about timings, recording strategy, editing, and publishing transcripts all shape the resulting product.^25^ At the same time, it enables collective remembering and dialogue, as well as registers disagreement on past events or their relative significance. In my experience on the story of Dolly the Sheep, curious dialogue about the relative biological importance of cloning from embryonic cells (Dolly's less famous precursors, Megan and Morag) versus Dolly being cloned from adult differentiated cells as a technical feat undermined some assumptions and pointed to informal debates not obvious in the published literature.

Oral historian's long‐standing concern with how memory is constructed in interviews and in groups is a valuable lesson in interpretation. Individual memory is notoriously unreliable, as those who have conducted interviews will know. Facts, dates, and order of events can easily be mixed up, and while some highlights are vividly remembered, other events of great interest to a historian may not have registered. When remembering, people often create coherence that wasn't necessarily there. Where interview evidence brings great value is in stories of personal relationships, depending on how willing one is to share them, but even more so in providing a glimpse into daily routines and practices, the kinds of knowledge and insight largely unavailable from published accounts of experiments or cases, and only occasionally present in archived correspondence or preserved laboratory notebooks.

What counts as the primary source in interpreting the conversation is not obvious, either. Indeed, the UK Oral History Society emphasises the recording as the primary source; a transcript is a useful but inferior document that can help with publishing and interfacing with the audio file, yet it cannot pick up the subtleties of intention, intonation, dialect, or mood.^26^ For social science research, by contrast, most of the work happens with the transcripts, even if recording and notes do play a role. But large‐scale anonymisation and use of software tools for coding interviews, such as NVivo, favour the transcript. Audio files can be redacted and anonymised, too, but that process is slow and laborious. It is possible to envision software solutions to these issues, and I suspect much could be learned from radio and podcast producers, but the centrality of text makes it more difficult in practice.

The challenges of working with memory and testimony that historians face are a useful reminder to extend the caution and caveats brought up with recorded testimony to other kinds of sources—not least critical approach to archival documents. Archives often retain the status of the historian's gold standard, going back to the nineteenth‐century debates that shaped Western history as a modern discipline, notably Leopold von Ranke's eminent insistence on prioritising (written) primary sources.^27^ Archives may be created, curated, and accessed in formalised ways, and are rightly cherished institutions, but what—and whose—materials are preserved is contingent on many ongoing decisions, and archives remain sites and conduits of power over identity and memory.^28^ Scientific and medical work, more specifically, also leaves a formalised record of publications, notoriously standardised in the recent past, and often opaque, yet even those could be read against the grain to reconstruct networks, tools, arguments, and timelines of publication. In most projects in the history of science, technology, and medicine, the research interview is one of the many sources a historian relies on and tries to make sense of.

## 5 Patchworks of Sources

Oral historians prioritise the interview corpus as not only deserving of recognition and use, but as the key source that can shed new light on people's lives and memories and bring insights not easily accessible from archival or published materials. They do, of course, also use printed and archival sources, but, subverting the common practice of doing history, these are not central. Some projects in history of science, technology, and medicine increasingly centre oral history as their main method.^29^ Yet most of us use interviews alongside other sources, from archives and published documents to film or Reddit posts; this was the case with all three projects discussed in this article. Oral testimony can add invaluable richness and unique narratives alongside the paper archive, but a full historical interpretation or causal argument would incorporate it among other sources. How one makes sense of the partial perspectives strewn across sources and prioritises across them remains a key challenge in the craft of historical work, and using oral testimony raises special concerns, because it looks back from the present and often brings in insights into the day‐to‐day practices and relationships not often found in the paper record. Here, building on Miguel García‐Sancho's call, I suggest that the metaphor of triangulation—used by social scientists and increasingly by historians—is helpful to arrive at a useful historical interpretation that remains connected to the patchwork of sources one manages to assemble.^30^

An anecdote I encountered in my training, used to illustrate how far the recognition of oral history as a valid method had come, looked back to interview guidelines written in the 1970s that emphasised securing documentary evidence, such as an individual's papers, as potentially the most valuable outcome of an interview. Now that the veneration of the archival document as the exemplary historical source may have weakened somewhat, and the interview is a respectable research tool, we can perhaps recognise that this is not entirely bad advice. Being able to access documents held in personal collections is a useful side effect of conducting interviews, if the interviewees are happy to share them. Even more productively, going through the documents during the conversation can be highly evocative and useful, both in bringing back memories and in highlighting the disconnect between what one remembers and what the documents say. Trying to resolve these gaps can shed light both on the testimony and on the story behind what is on the page.

For example, interviewing a retired civil servant was tremendously helpful for me, not only because of the fascinating and highly relevant memories, but also because of learning first‐hand how the civil service generated the papers that end up in the National Archives at Kew, what the conventions of address were, and how certain notes could be limited in their circulation. (Apparently, handwritten notes in pink ink would not be clearly reproduced by a photocopier in the 1980s, which had its value). The interview itself was built on my partial working through the relevant Home Office archives done to that point, shaping the conversation and adding new layers to the workings of the relevant government department. In addition, I was given extra documents I had not found in the national archives, which shed light on important issues regarding enforcement of guidelines.^31^

This special issue emphasises the work of interviews as “historical fieldwork,” which strikes me as very apt. A major benefit is the access to the community that interviewees can often provide, especially with the “snowball method” of finding them. Some interviews I have conducted where the rapport was lacklustre, or the interviewee had recounted the narrative many times in publications, and we did not move much beyond that, were nonetheless greatly useful for the connections they enabled. Sometimes, while the answers to questions may not always contribute to reframing the project, the act of conversation may change perspectives strongly. This is not necessarily limited to historical interviews—engagement with publics, patients, lay experts, or scientists in other contexts can have similar transformative effects. Moreover, the aspects outside of the actual interview—correspondence, conversations that are unrecorded, photographs, and shared documents—all contribute to the way a historian thinks about their questions and approach, even if these exchanges and ephemera do not always make it into a depository or onto the published page.

But what is one to make of what has been gathered? Triangulation is a metaphor for dealing with multiple sources that is used widely in the social sciences, adapted from geometry and surveying; a location of the point can be determined from known points through trigonometric calculations, relying on observed angles or known distances.^32^ Social research, with characteristic care it takes with methodology, distinguishes between different types of triangulations: across data and sources within a methodological framework, across investigators, across different methods, or across theoretical perspectives. There is some overlap between combining qualitative or quantitative approaches, also known as mixed methods research, although some theorists point to its epistemic pitfalls.^33^ Philosophers of science have similarly raised some concerns over issues of incomparability and discordance in the natural sciences when using different methods.^34^ As historians, perhaps we need not worry too much about typologies of methods or the intricacies of debates that do not straightforwardly map onto our idiosyncratic and patchy sources. The issue of discordance is not necessarily a problem either—quite the opposite. As Lillian Hoddeson persuasively demonstrated, disagreements between testimony and the archive can provide further opportunities for reflection, generate new questions, and interrogate both types of sources.^35^

In revisiting his formative 1970s work on triangulation in qualitative research, Norman Denzin has lamented its adoption into a positivist way of framing social science research, leaving qualitative insights subservient to quantitative methods, only there to provide extra texture.^36^ Instead, Denzin envisioned a need for *bricolage*, DIY, or tinkering with methods that seek to work across multiple perspectives and go between paradigms of social research (without fully synthesising them and recognising the difficulty of moving between them). This would lead one to provide explanations situated in individuals’ biographies and historical context, embrace the political potential of social research in transforming the present and confronting its many injustices, and to “turn history back against itself”.^37^ Much of this will resonate with many historians of science, technology, and medicine, whose work often points to the contingency of present systems of knowledge, prestige, and global power, and has continuously sought to bring in new actors and ways of thinking into these conversations. On a more modest level, however, working across sources while maintaining the critical traditions of interrogating them, without automatically assuming hierarchies of reliability but also without treating sources as interchangeable, can help pinpoint something valuable for a historian. Depending on one's epistemological convictions and ambitions, this might be a narrative, an account of an event, perhaps an approximation of a historical fact—but in any case, a rich interpretation.^38^

## 6 Conclusion

As this special issue demonstrates, research interviews continue to be a widespread and useful tool in the history of recent science, technology, and medicine, with scholars adapting various interview methodologies to suit the specific historical questions at hand. Yet, as this essay suggests, there is much to learn from increasingly diverse work being done by oral historians, particularly regarding the methodological interplay between interview techniques and in thinking about the relationship between interviewer and interviewee. My own work has focused less on life stories and more on gaining insights into professional practices, collective memories, and evolving narratives within the scientific community,^39^ but I have benefited greatly from training in oral history technique, the field's ethical expectations, and strategies for interpretation.

When it comes to reading interviews alongside other sources, moreover, the social sciences offer another source of inspiration. Triangulation across sources is crucial in writing history, and while we should not underestimate the value of testimony co‐constructed in an interview encounter, we can treat it within the collection of other sources we manage to compile and get to grips with. Historians routinely import and adapt theoretical perspectives from across different styles of doing history, but also from sociology, anthropology, geography, and philosophy, to name a few disciplines. Triangulation describes much work and sensibilities already present in history of science, technology, and medicine, and helps place the historical interview with its distinct aims and methods alongside other, more formalised ways of using conversation as a research tool.

Interviews allow historians to engage directly with living witnesses, creating a dynamic interplay between past and present. By embracing the methodological diversity and ethical complexity inherent in such work, historians can craft richer, more nuanced narratives that honour the multifaceted character of historical inquiry, while building relationships within the expert and lay communities they study. In this light, thinking of interviews as part of historical fieldwork feels appropriate. With that said, historians of science have spent much time elaborating and sometimes deconstructing the distinctions between the lab and the field, so perhaps some reflexivity is in order here, too.^40^ What are the distinctions between the library or archive and the historical field, and how do we choose to place and frame these within the disciplinary politics of our own communities?

## Conflict of Interest

The author declares no conflict of interest.

## Data Availability

The data that support the findings of this study are available from the corresponding author upon reasonable request.

## Footnotes

1 Some of this work was published as Myelnikov 2019; Myelnikov and García‐Sancho 2017; Myelnikov and Kirk 2022; Myelnikov 2024. For the Animal Research Nexus, see also Davies et al. 2020; Davies et al. 2024.

2 The UK's Oral History Society's current training opportunities are listed at https://www.ohs.org.uk/training/ (accessed 12 October 2025).

3
https://massobs.org.uk (accessed 12 October 2025).

4 See e.g. *An Oral History of The Office* podcast, hosted by Brian Baumgartner and produced by Propagate Content, 14 July–15 September 2020, available on Spotify, https://open.spotify.com/show/5T1519uHGVd6Te2xF5tKkR?si=43fc452d5d804619 (accessed 10 August 2025). For an extensive list, see Hunt 2015.

5
Vansina 1985; Miller 2011; Mahuika 2019.

6
de Chadarevian 1997.

7
Perks and Thomson 2016, on 136–46; see also Anderson and Jack [1991] 2016; Yow 1997.

8
Merchant 2019.

9
Flick 2018, on 83–94. For some counter‐examples, see Berkhout 2013; Akuffo 2023.

10
Thompson and Bornat 2017, on 337–342.

11 The 1989–1990 Bristol bombing targeted veterinary surgeons involved in laboratory animal welfare; see Vines 1990. One extreme case of criminal harassment happened in Staffordshire in 2004, when activists desecrated the grave of a guinea‐pig breeders’ mother‐in‐law and stole the remains.

12
Holmberg and Ideland 2010; Sharp 2018; Brandt et al. 2024.

13
Roe et al. 2024.

14 For a productive discussion of anonymity, and changing the decision mid‐way through the project, see Martin Hobbs 2021.

15
https://www.ohs.org.uk/legal‐and‐ethical‐advice/ (accessed 10 October 2025); British Library and Oral History Society 2019.

16
Bourke 2009.

17
Calabria and Bailey 2023.

18
Quinlan 2010; Perks and Thomson 2016, on 135–139.

19 See e.g. Anderson & Jack [1991] 2016; Yow 2016.

20
Hoddeson 2006; see discussion of the “mask” and “scientific persona” in this issue's introduction.

21 The distinction goes back to Oakeschott 1983. See also White 2014. For a critique of the historical vs practical past dichotomy, see e.g. Lorenz 2014.

22 For a clear mission statement, see preface to Shapin 2008.

23
Abir‐Am 1999 and other articles in the issue of *Osiris*. For the Wellcome Witness Seminar overview, see Tansey 2006.

24
Smith 2018.

25
Tansey 2006.

26
British Library and Oral History Society 2019, on 32. See also: Hutching 2004.

27
Grafton 1997, on 34–93; Eskildsen 2013; Paul 2013.

28
Schwartz and Cook 2002; Buchanan 2007; Powell 2018.

29
Snow 2020; Snow and Whitecross 2022; Duffy 2024.

30
García‐Sancho 2016. See also Donohue 2017.

31
Myelnikov 2024.

32
Denzin 1970; Denzin 2012; Flick 2018, on 527–544. *Consilience* is another term used by some historians, notably those who draw on evidence from the natural sciences and archaeology, for instance genomic data or ancient DNA, e.g. McCormick 2011. Going back to William Whewell's philosophy of science (Whewell 1858), and given new life again by E. O. Wilson on the 1990s (see Wilson 1998), consilience points to inductive inferences being stronger if several lines of evidence support them independently. The specific links to using the evidence from natural sciences—something that may sit especially uneasily with historians of science—makes it a less useful metaphor for the purposes of this article.

33
Denzin 2012.

34
Stegenga 2012; Kuorikoski and Marchionni 2016.

35
Hoddeson 2006.

36
Denzin 2012.

37 Ibid. On bricolage/tinkering in the laboratory sciences, see also Myelnikov 2019.

38 For a few positions in historiographic debates over historical interpretation, and connection between evidence and narratives that historians produce, see White 1973; White 2014; Evans 2018.

39 Of course, one might arrive at similar insights as part of a life stories approach.

40
Kohler 2002; Brinitzer and Benson 2022.
